# Geriatric Workforce Enhancement Programs' Participation in the Nursing Home COVID Action Network

**DOI:** 10.1093/geroni/igab046.1894

**Published:** 2021-12-17

**Authors:** Leland Waters, Nina Tumosa

## Abstract

In late September, 2020, the Geriatric Workforce Enhancement Program’s (GWEP) Program Officer, at the Health Resources Services Administration (HRSA), alerted the 48 GWEPs about a nationwide initiative focusing specifically on the pandemic’s effect in nursing facilities. The ECHO Institute at the University of New Mexico negotiated a national contract with the Agency for Healthcare Research and Quality (AHRQ) to provide a nationwide educational intervention via the CARES Act Provider Relief Fund. The ECHO Institute recruited over 100 Training Centers as educational coordinators for the Project ECHO Nursing Home National COVID Action Network. Our Project Officer suggested that individual GWEPs participate in this effort and take the lead or provide geriatric educators for these Training Centers. Project ECHO (Extension for Community Healthcare Outcomes) is an innovative telementoring program that creates virtual learning communities, bringing together healthcare providers and subject matter experts using videoconference technology for brief presentations, and case-based learning, fostering an “all learn, all teach” approach. This symposium will describe the journeys that five GWEPs experienced becoming Training Centers, rapidly deploying a nursing home ECHO project, to support nursing home staff on best practices for protecting patients, staff, and visitors from coronavirus infection and spread. GWEPs from The University of Louisville, the University of North Carolina, the University of North Texas, the University of Rochester and The Virginia Geriatric Education Center’s two ECHO Hubs, joined the National COVID Action Network. This presentation will provide an overview of why GWEPs are well positioned to address emergent needs with short notice.

